# A novel bone-thinning technique for transcranial stimulation motor-evoked potentials in rats

**DOI:** 10.1038/s41598-021-91780-5

**Published:** 2021-06-14

**Authors:** Yuyo Maeda, Takashi Otsuka, Takafumi Mitsuhara, Takahito Okazaki, Louis Yuge, Masaaki Takeda

**Affiliations:** 1 Department of Neurosurgery, Graduate School of Biomedical and Health Sciences, Hiroshima University, Kasumi 1-2-3, Minami-ku, Hiroshima, 734-8553 Japan; 2 Division of Bio-Environmental Adaptation Sciences, Graduate School of Biomedical and Health Sciences, Hiroshima University, Hiroshima, Japan

**Keywords:** Psychology, Neuronal physiology, Animal physiology, Neurology

## Abstract

Transcranial electrical stimulated motor-evoked potentials (tcMEPs) are widely used to evaluate motor function in humans, and even in animal studies, tcMEPs are used to evaluate neurological dysfunction. However, there is a dearth of reports on extended tcMEP recordings in both animal models and humans. Therefore, this study examined a new technique for stably recording tcMEPs over several weeks in six healthy female Sprague–Dawley rats. We thinned the skull bone using the skull base and spinal surgery technique to reduce electrical resistance for electrical stimulation. tcMEPs were recorded on days 1, 7, 14, 21, and 28 after surgery. The onset latency and amplitude of tcMEPs from the hindlimbs were recorded and evaluated, and histological analysis was performed. Stable amplitude and onset latency could be recorded over several weeks, and histological analysis indicated no complications attributable to the procedure. Thus, our novel technique allows for less invasive, safer, easier, and more stable extended tcMEP recordings than previously reported techniques. The presently reported technique may be applied to the study of various nerve injury models in rats: specifically, to evaluate the degree of nerve dysfunction and recovery in spinal cord injury, cerebral infarction, and brain contusion models.

## Introduction

Motor-evoked potentials (MEPs) are used to evaluate the function of the descending motor pathway by stimulating the motor cortex. Transcranial electrical stimulation MEP (tcMEP) is currently one of the most popular intraoperative pyramidal tract monitoring methods in clinical neurosurgery^1,2^, spinal surgery^3,4^, and aortic surgery^5^. Evaluating intraoperative motor function with tcMEPs often provides information on strategies to preserve postoperative motor function and evaluate patients following nerve injury (e.g., stroke or spinal cord injury)^6–8^.

tcMEPs have also been used in animal research to assess neurological dysfunction^9–12^. Some studies have stimulated the motor area from the surface of the skull using needle electrodes^9,10,12^, while others have stimulated the dura mater using microelectrodes^13^. Previous investigations have also recorded somatosensory evoked potentials over an extended period in rat models^10,14^. However, few reports have documented the use of tcMEPs for longitudinal evaluations in rat models^15,16^. While our laboratory has successfully recorded tcMEPs over time in mice with a brain contusion^17^, we found that achieving a sustainable and stable record of tcMEPs over several weeks was difficult when we attempted to record tcMEPs in rats over an extended period using previously reported techniques^15,17^. Focusing on the anatomical differences between rats and mice to identify the source of the difficulty, we speculated that the thicker and consequently increased electrical resistance of rat skulls could destabilize and compromise the electrical stimulation and extended tcMEP recordings of the rat brain. This study examined a new technique for stably recording tcMEPs via transcranial electrical stimulation in rats over several weeks that specifically addressed the increased thickness of the rat skull. We expect that the establishment of such an improved method could help improve the evaluation of the degree of nerve dysfunction and recovery in spinal cord injury, cerebral infarction, and brain contusion models.

## Results

### Electrophysiological recording

**Figure 1:**
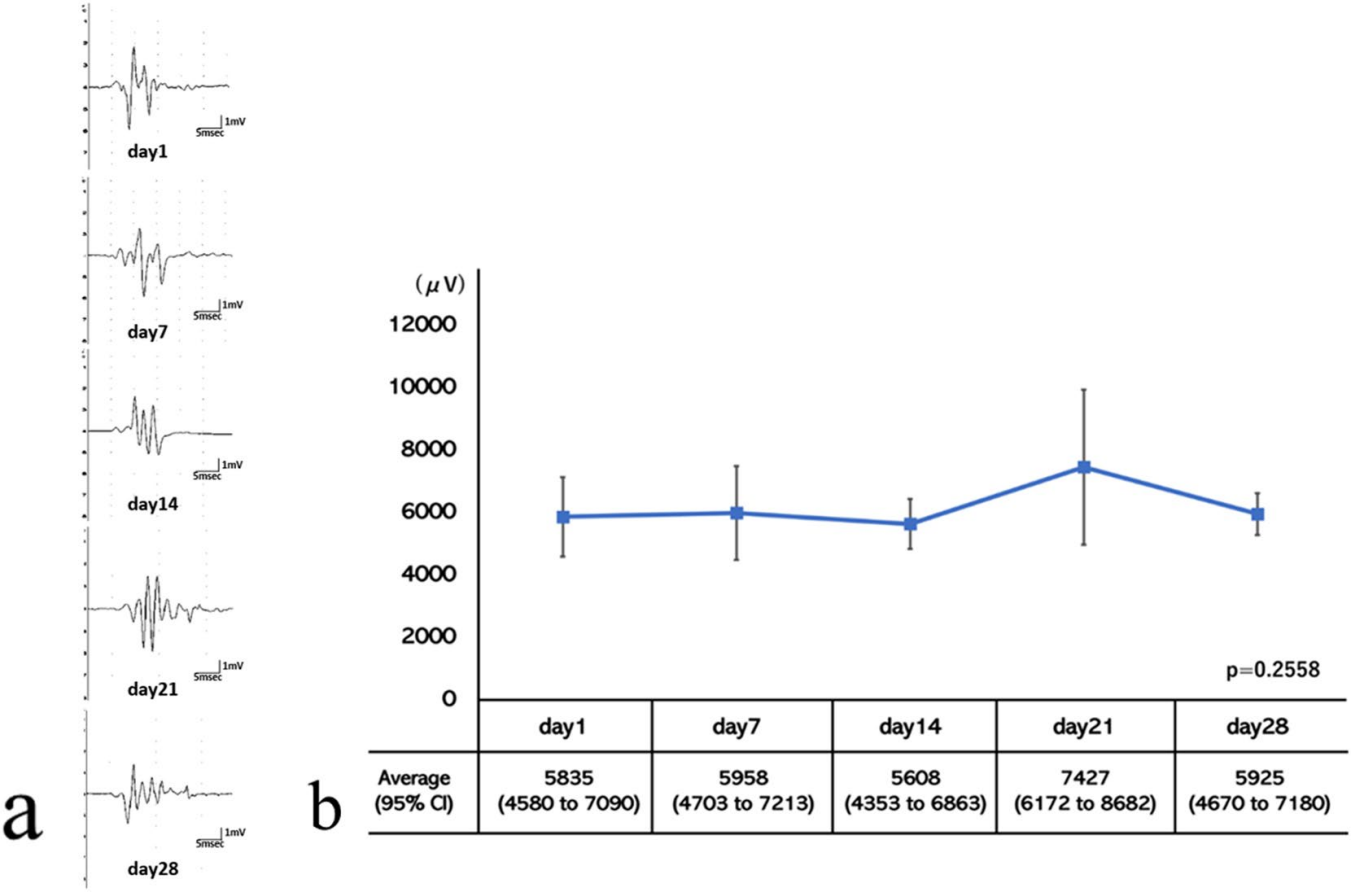
(**a**) Representative recordings of transcranial electrical stimulated motor-evoked potentials in the hindlimbs of female Sprague–Dawley rats plotted at 7, 14, 21, and 28 days after surgery. (**b**) Time course of the motor-evoked potential amplitude. Repeated measures analysis of variance was performed on the weekly measurements. *P*-value = 0.2558. CI = confidence interval.

Representative tcMEPs recorded at 1, 7, 14, 21, and 28 days after surgery are shown in Fig. 1a. The mean tcMEP amplitude was 5835 ± 1270 μV at day 1, 5958 ± 1501 μV at day 7, 5608 ± 800 μV at day 14, 7427 ± 2485 μV at day 21, and 5925 ± 668 μV at day 28. The tcMEP amplitude neither decreased nor increased significantly across time (*p* = 0.2558). The lower and upper limits of the 95% confidence interval were 4580 to 7090 μV on day 1, 4703 to 7213 μV on day 7, 4353 to 6863 μV on day 14, 6172 to 8682 μV on day 21, and 4670 to 7180 μV on day 28. The results of the weekly analysis of variance and the transition of the 95% confidence interval suggested that a stable amplitude could be measured during our study (Fig. 1b).

**Figure 2:**
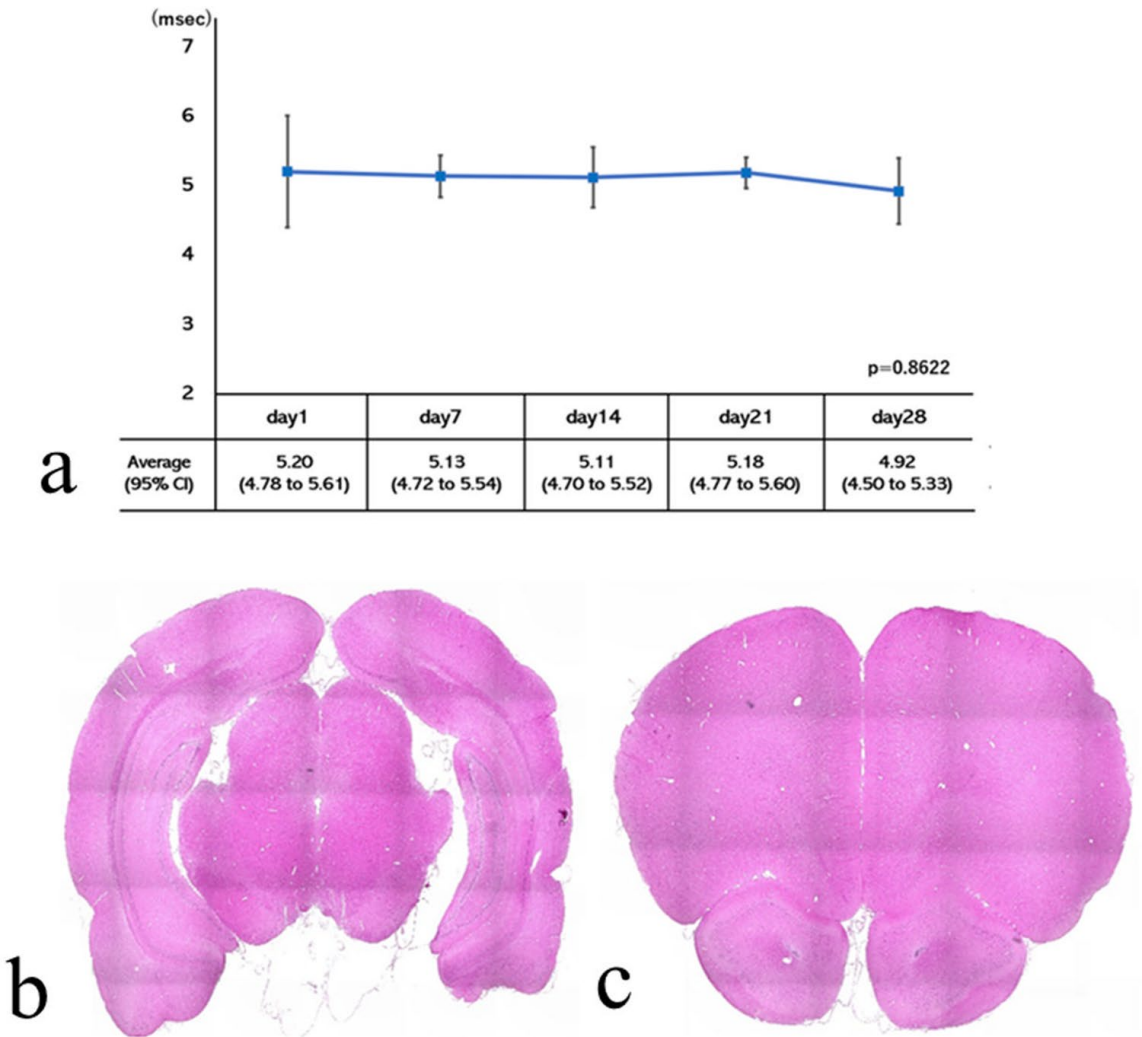
(**a**) Time course of the latency of transcranial electrical stimulated motor-evoked potentials. Repeated measures analysis of variance was performed on weekly measurements. *P*-value = 0.8622. CI = confidence interval. (**b**) A pathological image of a coronal section of the frontal lobe, located directly under the bregma. (**c**) Coronal section of the parietal lobe located directly under the lambda. There was no cerebral contusion or atrophy associated with this experiment.

The mean onset latency was 5.20 ± 0.80 ms on day 1, 5.13 ± 0.30 ms on day 7, 5.11 ± 0.45 ms on day 14, 5.18 ± 0.43 ms on day 21, and 4.91 ± 0.47 ms on day 28. No significant differences were observed between time points (*p* = 0.8622). The lower and upper limits of the 95% confidence interval were 4.78 to 5.61 ms on day 1, 4.72 to 5.52 ms on day 7, 4.70 to 5.52 ms on day 14, 4.77 to 5.60 ms on day 21, and 4.50 to 5.33 ms on day 28. The results of the weekly analysis of variance and the transition of the 95% confidence interval suggested that a stable latency could also be measured during our study (Fig. 2a). The latency and amplitude in the waveform were stable over time.

### Histological analysis

The site of transcranial electrical stimulation for tcMEPs over time was histologically analyzed. Both the cell nucleus and layer structure of the cerebral cortex were maintained. No formation of cavity and gliosis indicative of damage to brain tissue was observed. These findings indicate that our experiment did not cause brain damage (Fig. 2b, c).

## Discussion

The results of our study indicate that the bone-thinning technique enables the successful recording of tcMEPs of the hindlimbs in healthy rats over a month. Our data show that the latency and amplitude in the waveform were stable over time, and tcMEPs can be recorded stably and safely. Several studies have used tcMEPs as a method to evaluate motor pathways and the neurological recovery of brain contusion and spinal cord injury in rats^10,15,18–21^. tcMEPs can be elicited using needle electrodes inserted subdermally^10^, a corkscrew-type needle electrode inserted on the skull^18^, or a silver-disk electrode on the dura mater at the burr hole^19^. However, in tcMEPs recorded over time, reducing the invasiveness of the model at the time of each recording is essential. The use of percutaneous needle electrodes for stimulation and recording is a minimally invasive and simple procedure, allowing for serial electrophysiological testing in the same animal over a longer time. In this study, we only inserted the electrical needles for each recording, so sustainable electrode placement was not required, indicating that our technique is both less invasive and simple.

There is a dearth of reports on the techniques for recording tcMEPs over extended periods in rats. While there are a few reports on tcMEP recordings over short periods from hours to days^10,19^, there are even fewer reports on longer periods across several weeks^15,16^. The reason for this is the difficulty of extended tcMEP recordings. Therefore, there is a need for a technique that makes tcMEP recordings over time easier and more stable.

**Figure 3:**
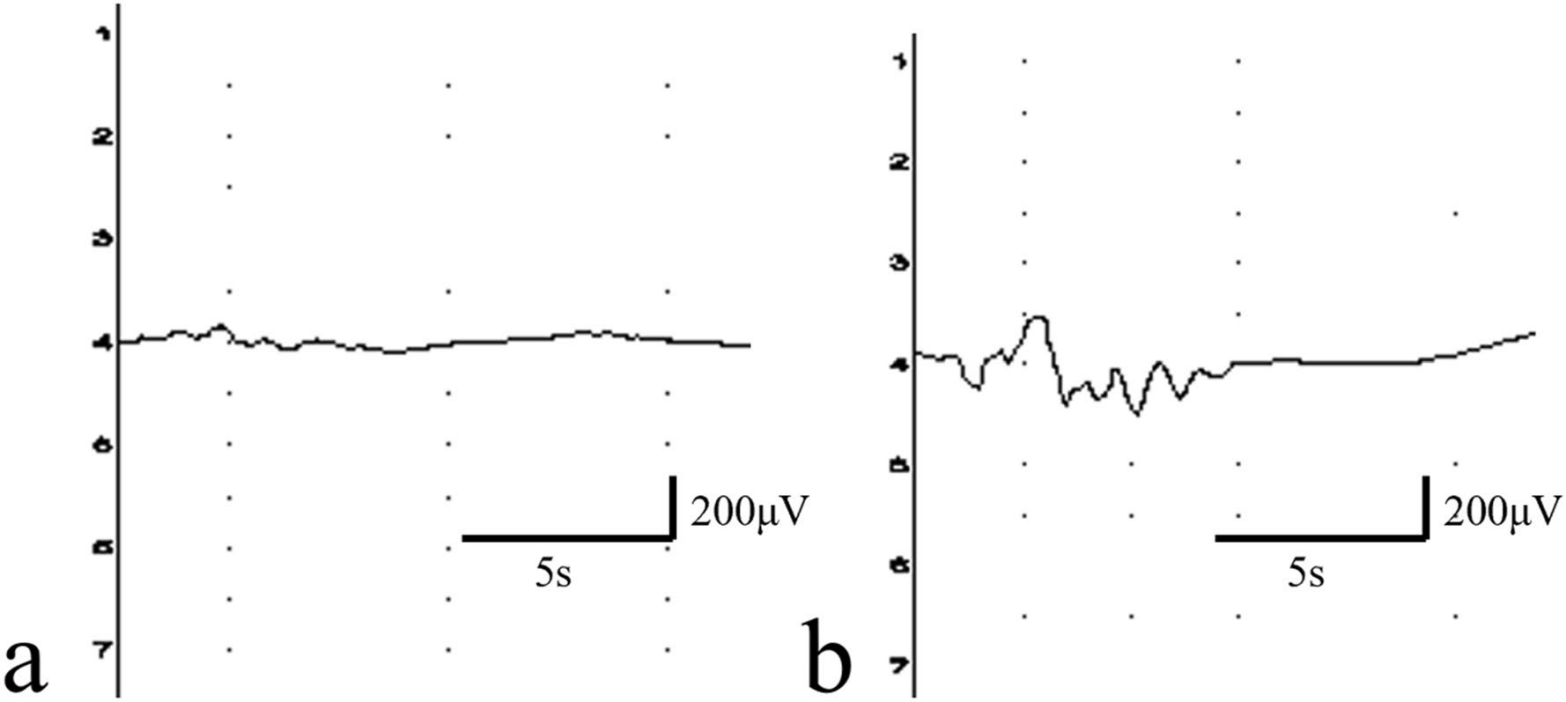
The waveforms of transcranial electrical stimulated motor-evoked potentials (tcMEPs) recorded using a previous technique (**a**) No tcMEPs were derived. (**b**) Only unstable waveforms were observed.

In previous reports, tcMEPs of the forelimbs^16^ and the hindlimbs^15^ of rats could be recorded over ≥ 1 week using subdermally inserted needle electrodes. In addition, our presently reported data may be useful for investigating changes in tcMEPs over time in mice models of central nervous system (CNS) injury. A technique for recording tcMEPs of the hindlimbs in mouse models of brain contusion over several weeks using subdermal needle electrodes has been previously reported^17^. However, when we attempted to use these previously reported techniques^15,17^ in rats, we found the stable and repeated recording of tcMEPs in the hindlimbs to be very difficult (Fig. 3a, b). We then hypothesized that the rat skull was thicker than that of the mouse, resulting in a high electrical resistance value for cortical stimulation so that the motor cortex could not be stimulated stably. Therefore, we created a bone-thinned model to reduce the resistance in transcranial electrical stimulation. Here, we recorded stable and repeated tcMEPs, suggesting that the decrease in electrical resistance for transcranial stimulation may contribute to more stable tcMEP recordings. Additionally, transcranial electrical stimulation mostly flows outside the skull due to the high electrical resistance of the skull, and only 10% of this stimulation reaches the brain^22^. Therefore, strong transcranial electrical stimulation induces foramen magnum stimulation, which in turn stimulates the medulla oblongata^23^. Since our method reduced the electrical resistance of the skull, a relatively weak stimulation of the motor cortex without that of the medulla oblongata may be sufficient to record tcMEPs. This indicates that our model may enable a more stable recording of tcMEPs than previous techniques, requiring only subdermal needle electrode insertion. Although a few existing reports^15,16^ have recorded tcMEPs over time in spinal cord injury models, there are no reports showing the course of tcMEPs in models without CNS injury. In this study, we succeeded in recording tcMEPs over time in healthy rats. Our data show that the amplitude and latency in the waveforms of tcMEPs were stable and constant over time, unaffected by repeated transcranial electrical stimulations and individual growth. These data will also be helpful to analyze the data from tcMEP recordings over time in previous reports.

The technique of scraping the skull of rats in a procedure similar to ours has been previously reported; however, in the previous procedure, all the layers of bone were shaved, and the electrodes were placed directly on the dura mater^13^. Therefore, some rats demonstrated subarachnoid hemorrhage or brain contusion as complications. In our technique, we left the thin inner wall of the skull bone after thinning the bone; therefore, there were no complications such as subarachnoid hemorrhage or contusions. Our histological analysis also showed no complications due to the procedure. Thus, our technique might be safer and more reproducible than previously reported techniques.

Recently, behavioral evaluation scales have been used as indicators of neurological recovery in animal models of CNS injury. The modified neurologic severity score^24–26^, the Basso, Beattie and Bresnahan locomotor scale method^6,10,11,27–29^, angle of inclined plane test^10,11,28,29^, and the Rotarod test^30–32^ have been used as behavioral evaluation scales. While it is true that these behavioral evaluation scales can be measured easily, detailed quantification is difficult, whereas the evaluation of tcMEPs is quantified. Although the amplitude of tcMEPs depends on the continuation of neuronal connection and the number of fibers regenerated, which plays a role in conduction, tcMEPs cannot evaluate specific behavioral differences in the process of nerve recovery. This means that tcMEP can be a complementary evaluation to behavioral evaluations, especially in CNS injury models. Notably, combining tcMEPs and behavioral evaluation scales may contribute to more detailed observations and elucidation of the process of nerve function recovery, and therefore longitudinal evaluation of tcMEPs is required for comparison with motor function evaluation over time. However, at present, few reports have compared and evaluated tcMEP recording and behavioral evaluation scales over a long period due to the difficulty of extended tcMEP recordings. Our technique enables easier and more stable tcMEP recording of the hindlimbs over a month than previously reported techniques. Therefore, our novel technique can be a breakthrough that solves the difficulty of extended tcMEP recordings. Specifically, regarding the applicability of tcMEPs to CNS injury models, it is necessary to record tcMEPs of the hindlimb instead of the forelimb—especially in paraplegia models, such as injury models of the thoracolumbar spinal cord. Thus, this technique is suitable for studies investigating paraplegia due to cerebral and spinal injury.

Our study has some limitations. Our research design was a prospective experimental animal study to create and evaluate a rat model that allows a more stable recording of tcMEPs over time compared to previous techniques. Further, this study had a small sample size. In the future, studies with larger sample sizes and longer follow-up periods are needed to generalize the results and gain insight into the long-term impacts of thinning skulls in rats. Furthermore, we would like to apply this technique of extended tcMEP recordings to elucidate the therapeutic mechanisms, such as cell therapy, drug therapy, and rehabilitation effect, in CNS disorder models such as spinal cord injury, brain contusion, and cerebral infarction.

In conclusion, the presently reported model is a relatively non-invasive, easy, and stable technique of extended tcMEP recordings. Using this technique may allow for repeated assessment of the degree of neurological dysfunction and the long-term quantification of dysfunction. Hence, our technique may be applied to the investigation of various nerve injury models in rats.

## Methods

### Ethics statement

All methods are also reported in accordance with ARRIVE guidelines (https://arriveguidelines.org), approved by the institutional ethical review committees (the Animal Experiment Committee of Hiroshima University, Hiroshima, Japan.), and conducted under the authority of the Project Licence (A19-73).

### Animals and surgical procedure

This study used six female adult Sprague–Dawley rats (Charles River, Kanagawa, Japan), with a mean weight of 273 g (range 250–300 g). We created the maximum number of models possible during this study. Referring to previously published reports that have recorded tcMEPs in similar numbers of animals^19,26^, we deemed our sample size sufficient to evaluate our model.

**Figure 4:**
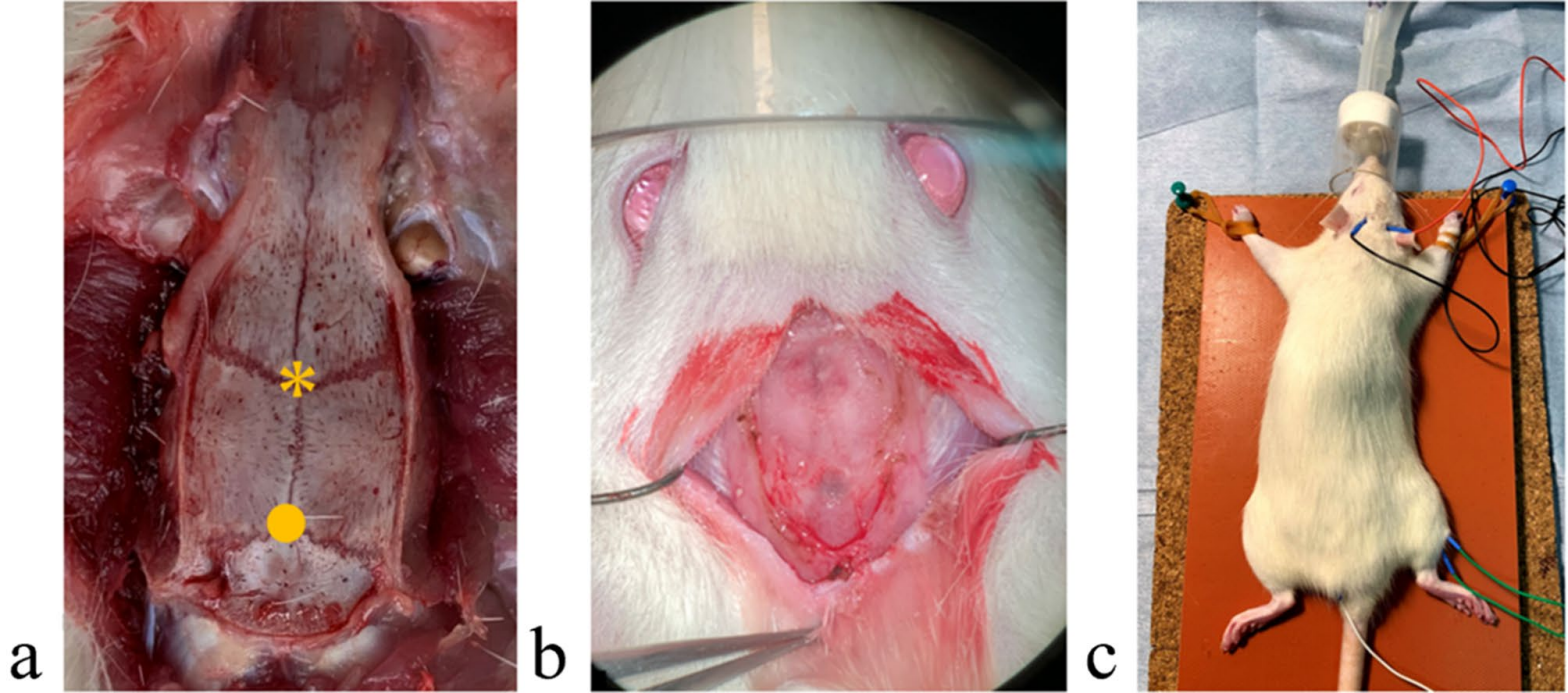
(**a**) Anatomical image of the rat skull seen from above. The asterisk indicates the bregma, and the circle marks the lambda. (**b**) Intraoperative image: skull thinning for a total diameter of 5 mm was performed. The outer plate and diploe of the skull were removed, leaving only the inner plate. The anode was installed on the lambda, and the cathode was installed on the bregma. (**c**) The electrode installation. Transcranial electrical stimulated motor-evoked potentials were recorded under general anesthesia.

Inhalation anesthesia was induced with 1.5% isoflurane, and the rats’ average body temperature was maintained at 28.0 °C. An incision was made to expose the skull, and then the skull was thinned (total area diameter = 5 mm) with a diamond drill at the bregma and lambda positions (Fig. 4a, b). We thinned the bone using the technique described by Mizuno et al*.*^33^. This thinning bone technique was used to create a low electrical resistance area for optimal electrical motor area stimulation. Transcranial electrical stimulation was performed through the thinned skull position using needle electrodes.

### Electrophysiological recording

For stimulations, two monopolar needle electrodes (Natus, Middleton, WI) were used. The anode was installed on the lambda, and the cathode was installed on the bregma. Transcranial electrical stimulation was performed with a train of four stimuli administered across a total duration of 0.5 ms through the subdermal needle electrodes. Supramaximal stimulation was used, and the average value of the stimulation was 72 V. The tcMEP was recorded from the needle electrodes inserted into the belly of the quadriceps femoris in the rat’s hindlimb (Fig. 4c). The stimulation and recordings were performed using Endeavor CR (Nicolet Biomedical, Madison, WI). tcMEP was recorded on days 1, 7, 14, 21, and 28 after surgery. Onset latency and amplitude were recorded and evaluated. The monopolar needles were inserted before every recording. This technique allowed the recording of tcMEPs of both the forelimbs and hindlimbs on both sides using this technique; however, we focused on evaluating tcMEP of the hindlimbs.

### Histological analysis

On day 28, the rats were anesthetized as previously described, and their brains were removed after perfusion with 4% paraformaldehyde in phosphate buffer. Brains were embedded in paraffin and cut into 5-μm-thick coronal sections using a cryostat (Leica Microsystems GmbH, Wetzlar, Germany). The segments were mounted on microscope slides to be used for hematoxylin and eosin (H&E) staining for histological analyses. The H&E-stained segments were examined under a multifunctional microscope (BZ-9000; KEYENCE Co., Osaka, Japan) to investigate cerebral contusion and atrophy associated with both bone thinning and electrical stimulation.

### Statistical analysis

Continuous variables are expressed as mean ± SD. Weekly continuous variable comparisons were performed using repeated measures one-way analysis of variance. A *p*-value of < 0.05 was considered statistically significant, and all statistical analyses were performed using JMP software V.12.

## Data Availability

The raw data supporting the findings of this study are displayed in Figs. 1, 2, 3 and 4. The authors declare that all data supporting the findings of this study are available within the paper.
